# Correction: Summary of Glaucoma Diagnostic Testing Accuracy: An Evidence-Based Meta-Analysis

**DOI:** 10.14740/jocmr2643wc1

**Published:** 2017-01-25

**Authors:** Saad Ahmed, Zainab Khan, Francie Si, Alex Mao, Irene Pan, Fatemeh Yazdi, Alexander Tsertsvadze, Cindy Hutnik, David Moher, David Tingey, Graham E. Trope, Karim F. Damji, Jean-Eric Tarride, Ron Goeree, Omar Akhtar, William Hodge

**Affiliations:** a Ivey Eye Institute, University of Western Ontario, London, ON, Canada; b Faculty of Medicine, Queen’s University, Kingston, ON, Canada; c Ottawa Hospital Research Institute, University of Ottawa, Ottawa, ON, Canada; d Department of Ophthalmology and Visual Sciences, University of Toronto, Toronto, ON, Canada; e Department of Ophthalmology, University of Alberta, Edmonton, AB, Canada; f Program for the Assessment of Technology and Health (PATH), and Department of Clinical Epidemiology and Biostatistics, McMaster University, Hamilton, ON, Canada; g Department of Epidemiology and Biostatistics, University of Western Ontario, London, ON, Canada

Corrections to article “Summary of Glaucoma Diagnostic Testing Accuracy: An Evidence-Based Meta-Analysis”, by Saad Ahmed et al, published in Vol. 8, No. 9, 2016, p641-649, doi: http://dx.doi.org/10.14740/jocmr2643w. The authors of this article would like to add an author, Omar Akhtar, who is entitled to authorship, the new author’s list should read as follows.

Saad Ahmed^a^, Zainab Khan^b^, Francie Si^a^, Alex Mao^a^, Irene Pan^a^, Fatemeh Yazdi^c^, Alexander Tsertsvadze^c^, Cindy Hutnik^a^, David Moher^c^, David Tingey^a^, Graham E. Trope^d^, Karim F. Damji^e^, Jean-Eric Tarride^f^, Ron Goeree^f^, Omar Akhtar^g^, William Hodge^a, g, h^

^a^Ivey Eye Institute, University of Western Ontario, London, ON, Canada

^b^Faculty of Medicine, Queen’s University, Kingston, ON, Canada

^c^Ottawa Hospital Research Institute, University of Ottawa, Ottawa, ON, Canada

^d^Department of Ophthalmology and Visual Sciences, University of Toronto, Toronto, ON, Canada

^e^Department of Ophthalmology, University of Alberta, Edmonton, AB, Canada

^f^Program for the Assessment of Technology and Health (PATH), and Department of Clinical Epidemiology and Biostatistics, McMaster University, Hamilton, ON, Canada

^g^Department of Epidemiology and Biostatistics, University of Western Ontario, London, ON, Canada

^h^Corresponding Author: William Hodge, Ivey Eye Institute, University of Western Ontario, 268 Grosvenor St, London, ON N6A 4V2, Canada

